# Molecular Cloning, Bioinformatics Analysis and Expression of Insulin-Like Growth Factor 2 from Tianzhu White Yak, *Bos grunniens*

**DOI:** 10.3390/ijms15010504

**Published:** 2014-01-03

**Authors:** Quanwei Zhang, Jishang Gong, Xueying Wang, Xiaohu Wu, Yalan Li, Youji Ma, Yong Zhang, Xingxu Zhao

**Affiliations:** 1 College of Veterinary Medicine, Gansu Agriculture University, Lanzhou 730070, China; E-Mails: quanweizhang@st.gsau.edu.cn (Q.Z.); gongjishang@st.gsau.edu.cn (J.G.); xueyingwang403@gmail.com (X.Wa.); wx.258.h@st.gsau.edu.cn (X.Wu); liyalan0819@gmail.com (Y.L.); zhangyong@gsau.edu.cn (Y.Z.); 2 College of Animal Science and Technology, Gansu Agriculture University, Lanzhou 730070, China; E-Mail: yjma@gsau.edu.cn

**Keywords:** Tianzhu white yak, *IGF2* gene, rapid amplification of cDNA ends (RACE), sequence analysis, bioinformatics

## Abstract

The IGF family is essential for normal embryonic and postnatal development and plays important roles in the immune system, myogenesis, bone metabolism and other physiological functions, which makes the study of its structure and biological characteristics important. Tianzhu white yak (*Bos grunniens*) domesticated under alpine hypoxia environments, is well adapted to survive and grow against severe hypoxia and cold temperatures for extended periods. In this study, a full coding sequence of the *IGF2* gene of Tianzhu white yak was amplified by reverse transcription PCR and rapid-amplification of cDNA ends (RACE) for the first time. The cDNA sequence revealed an open reading frame of 450 nucleotides, encoding a protein with 179 amino acids. Its expression in different tissues was also studied by Real time PCR. Phylogenetic tree analysis indicated that yak IGF2 was similar to *Bos taurus*, and 3D structure showed high similarity with the human IGF2. The putative full CDS of yak *IGF2* was amplified by PCR in five tissues, and cDNA sequence analysis showed high homology to bovine *IGF2*. Moreover the super secondary structure prediction showed a similar 3D structure with human IGF2. Its conservation in sequence and structure has facilitated research on IGF2 and its physiological function in yak.

## 1. Introduction

Yak (*Bos grunniens*) is the most important animal species in the Tibet plateau not only because of its economic value in meat, milk, leather and velvet production [1], but also as an ideal model animal for studying the adaption mechanisms in a cold, low oxygen environment. The insulin/IGF/relaxin superfamily is an ancient, functionally diverse protein family functioning in metabolism, growth and differentiation. Insulin or insulin-like proteins have been described in unicellular eukaryotes as well as in primitive species such as insects, tunicates, annelids, and molluscans [2]. Insulin-like growth factors (IGFs) are important members of the growth hormone-insulin-like growth factor-1 axis (GH-IGF1), and play important roles in fetal growth, development, proliferation, differentiation, apoptosis and transformation, especially in mammalian muscle [3,4] and cartilage cells [5]. IGFs contain the two best-characterized members, IGF1 and IGF2 [6]. Both of them, together with insulin, are members of a family of small proteins that show similarity at the levels of primary and tertiary structure, and some overlap in biological activities [7,8]. IGF1 and IGF2 are responsible for the growth and development of somatic tissues, such as skeletal muscle and bone [9]. And IGFs also exert an anabolic effect in carbohydrate metabolism by stimulating glucose transport and glycogen deposition, and in protein metabolism by stimulating amino acids uptake, protein synthesis, and inhibiting proteolysis [10]. Evidence has suggested that both IGF1 and IGF2, by binding to their specific, high affinity membrane receptors, can activate multiple signal transduction pathways, which turn on muscle-specific genes and facilitate protein synthesis of myofibrils [11]. IGF axis components produced locally, including IGF1, IGF2, their binding proteins (IGFBPs 1 to 6) and their receptors, play a key role in longitudinal bone growth, as shown by *in vitro* and *in vivo* experiments [12]. Disruption of IGF1 or IGF2 signaling results in prenatal growth retardation [13]. Several data *in vitro* have shown expression of both IGFs in the growth plate [12,14], while some studies *in vivo* have yielded conflicting results, with IGF2 being the most abundant, whereas IGF1 could only be detected depending on animal ages or techniques used [15,16].

The growth in yak has been limited by many factors, because it lives in the environment of high attitude, low oxygen content, short period of grass growth, cold and adverse conditions, but can adapt to the ecological environment of the cold alpine pastoral areas well. As far as we know, reports about endogenous factors influencing yak growth are rare, and no study has investigated the yak *IGF2* gene and its exact functions. The main aim of the present study was to obtain the full-coding sequence corresponding to *IGF2* and predict its structure and role in yak. Our study indicated the existence of yak *IGF2* for the first time and its potential roles in yak performance, which is important for a better understanding of its bone and skeletal muscle growth and differentiation. A more comprehensive understanding of IGF2 actions in muscle will undoubtedly contribute to the understanding of basic growth physiology of vertebrates in general and may also have important implications in muscle hypertrophy, muscle atrophy, and muscle regeneration.

## 2. Results and Discussion

### 2.1. Result

#### 2.1.1. Cloning and Characterization of the Full Coding Region of the Yak *IGF2* Gene

**Figure 1.**
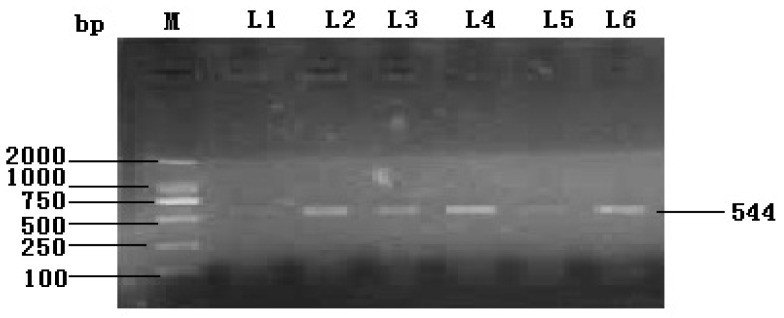
PCR products of IGF2 gene coding sequence in Tianzhu white yak. **M**, marker, **L1**, **L2**, **L3**, **L4**, **L5** and **L6** stand for brain, kidney, spleen, liver, ovary and muscle, respectively.

**Figure 2.**
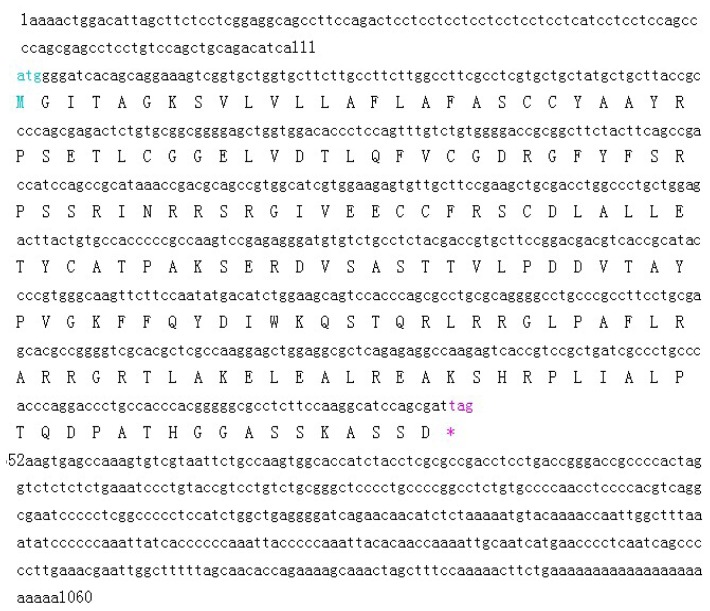
Complete nucleotide sequence encoding *Bos grunniens* IGF2 and deduced amino acids of the cloned IGF2. The sequences were submitted to NCBI GenBank (accession number KF682139). * was the termination codon.

Based on sequence homology, the designed primers amplified a single 544 bp fragment (Figure 1). RACE technology was used and successfully obtained a full-length cDNA fragment of 1060 bp. The sequence of *IGF2* gene analysis showed a 540 bp open reading frame (ORF), and 111 and 409 bp corresponding to the 5′- and 3′-terminal UTR (untranslated regions) respectively (Figure 2). The sequence was submitted to NCBI GenBank (acccession number KF 682139). The sequence indicated a length of 1060 bp, and covered the full coding region comparing with the corresponding regions from different organisms. The deduced amino acid sequence of IGF2 was found to consist of an open reading frame of 179 amino acid residues (Figure 2). The BLAST analysis for the coding region of yak IGF2 showed that it shared high similarity (100%–88%) with IGF2 from other mammals (100% *Bubalus bubalis*, 99% *Bos taurus*, 98% *Ovis aries*, 98% *Cervus elaphus*, 96% *Cervus nippon*, 90% *Canis lupus*, 89% *Sus scrofa*, 88% *Homo sapiens*, 88% *Pan paniscus* and 88% *Nomascus leucogenys*).

#### 2.1.2. Amino Acid Composition and Protein Secondary Structure

The molecular analysis of the 179 amino acid sequence of yak IGF2 using the program PROTEAN [17] showed that this protein has a molecular weight of 19.68 kDa and pI 8.82. The predicted protein contained 63 hydrophobic residues (35.20%), 18 acidic residues (10.10%), 25 basic residues (13.97%) and 49 polar amino acids (27.37%). The instability index (II) was computed to be 59.56. Aliphatic index and grand average of hydropathicity (GRAVY) of yak IGF2 was 78.04 and −0.184, respectively. The total number of negatively charged residues (Asp and Glu) was 18, and the total number of positively charged residues (Arg and Lys) was 25. These data suggest that the yak IGF2 protein is an unstable protein. The *N*-terminal of the sequence was considered M (Met) and the chemical composition of the predicted protein is illustrated in Table 1.

#### 2.1.3. Multiple Sequence Alignment

**Figure 3.**
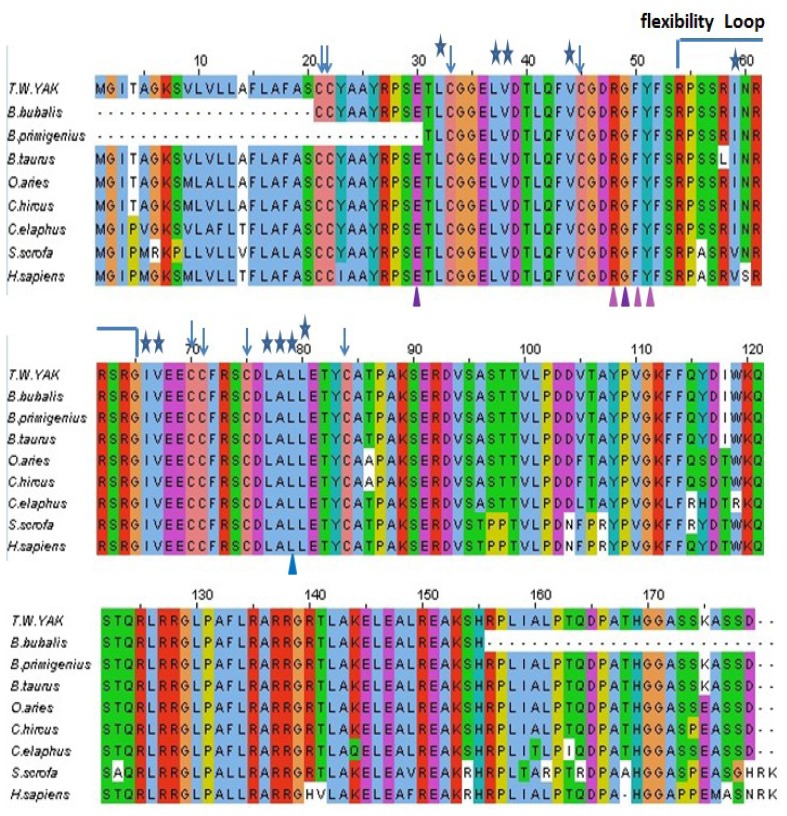
IGF2 amino acid sequence of the yak was compared with those from eight different mammalian proteins. The alignment was generated with the MAFFT (Multiple Alignment using Fast Fourier Transform) Multiple Sequence Alignment program. Residues were color coded according to their conservancy. The highly conserved IGFBP binding residues were labeled by (violet Δ), IGFBP and IGF-receptor and insulin-receptor binding residues were labeled by (lilac Δ) and IGF-receptor binding residues were labeled by (blue Δ). Conserved residues facilitate the binding of IGFBP, IGF- and insulin-receptor and IGF receptor. Conserved cysteines are shown as ↓. The hydrophobic residues forming the binding interface are shown as (blue ⋆). There was a constant flexibility loop between the binding sites.

The amino acid sequence of yak IGF2 was aligned with that of eight mammalian species by ClustalW [18,19] (Figure 3). The disulfide bond formed by these cysteines (Cys 21, Cys 22, Cys 33, Cys 45, Cys 70, Cys 71, Cys 75 and Cys 84) were highly conserved in all compared proteins, and responsible for the stability of IGF2. Yak IGF2 was mainly a single chain protein with three domains that existed different binding sites, which played a structural and catalytic role in IGF, compared with that of different mammalian species. There were three binding surfaces in the IGF2 amino acid sequence, of which the first was the IGFBP-binding surface from Glu-30 to Tyr-51 with α-helix, the second was IGF-receptor binding and insulin-receptor binding surface from Phe 52 to Arg 64 with α-helix, and the third was IGF-receptor binding surface with two sites (Gly 65 and Ala 85). The stable mammalian IGFs were held together mainly by conserved hydrophobic residues (Figure 3).

**Figure 4.**
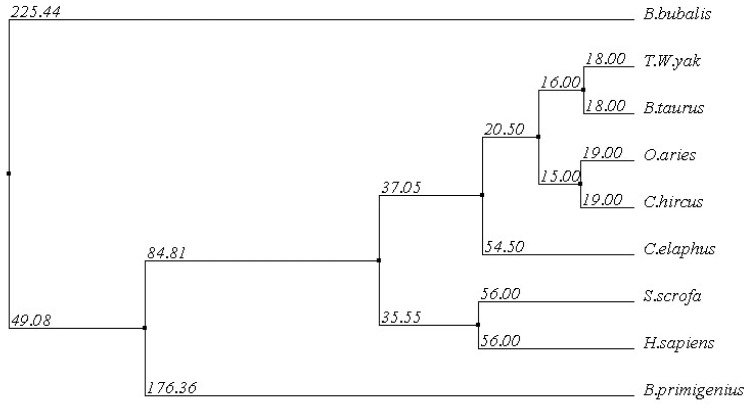
The phylogenetic tree of yak IGF2 and potentially related genes. The protein sequence of yak IGF2 was compared with other mammalian sequences of the GenBank data base. The alignment was generated with the BLOSUM 62 from MAFFT Multiple Sequence Alignment.

**Figure 5.**
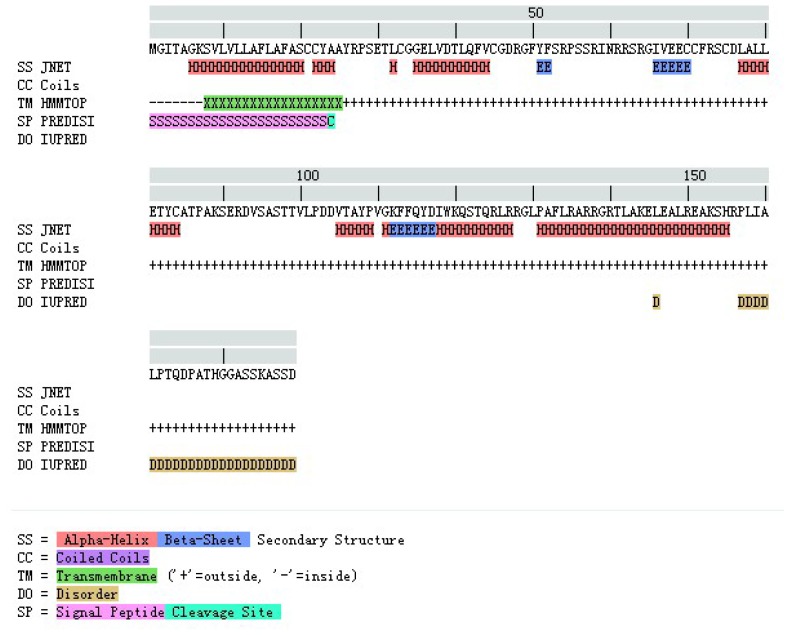
The secondary structure annotation sites of the yak IGF2 sequence using Bioinformatics Toolkit Quick2D program.

The comparison between the predicted amino acid sequence of yak IGF2 and the sequences from the best characterized representatives of IGF2 from different organisms was carried out. The BLASTP analysis showed that yak IGF2 shared high similarity with IGF2 from different mammalian species. y p g The amino acid sequence of white yak IGF2 was 100% similarity with *Bos primigenius* and *Bos bubalis*, 99% with *Bos taurus*, 96% with *Ovis aries* and *Capra hircus*, 92% with *Cervus elaphus*, 84% with *Sus scrofa* and *Homo sapiens*, respectively (Table 2 and Figure 3). Such high similarity proposed a close evolutionary relationship. The phylogenetic tree of the examined proteins indicated that yak IGF2 groups with *Bos taurus* (Figure 4). A prediction of the secondary structure analysis of yak IGF2 was carried out using Bioinformatics Toolkit Quick2D program (Figure 5). The predicted structure suggested that this protein was composed of seven helixes.

#### 2.1.4. 3D Structure Prediction

**Figure 6.**
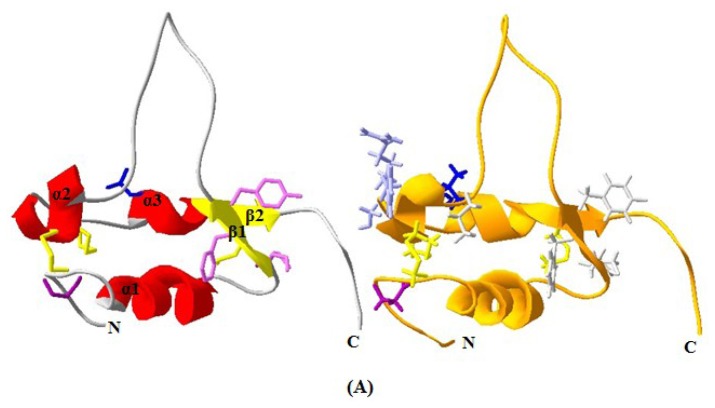

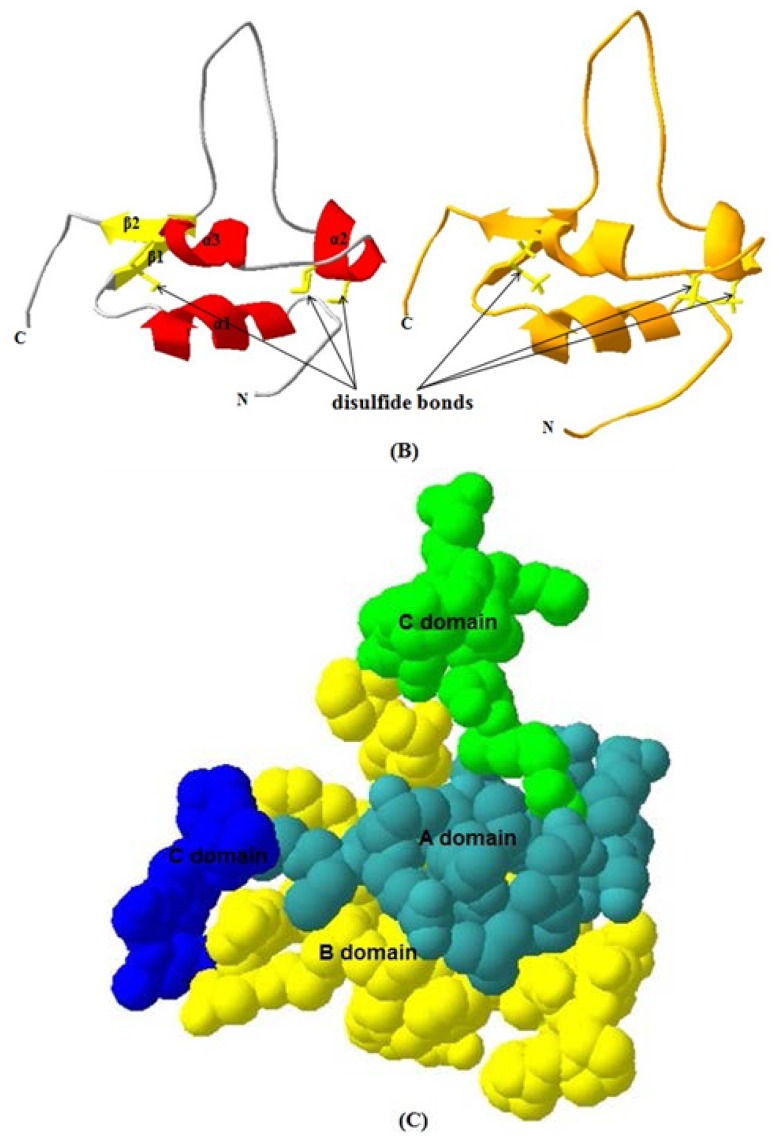
Predicted 3D structure model of yak IGF2 and the mainly chain interface structure. The 3D structure model was predicted by Swiss-model server. Left: yak IGF2, right: human IGF2. (**A**) Each chain contains 3 helixes. Binding sites were shown as rodlike molecules for yak IGFBP (modena), yak IGFBP and IGF-receptor and insulin-receptor (mauve) and yak IGF-receptor (blue); human IGFBP (modena), type 1 human IGF-receptor and insulin-receptor (white), type 2 human IGF receptor and IGFBP (French grey), human IGF-receptor (blue); (**B**) The view of IGF2 reveals three disulfide bonds (yellow, rod-like) which stabilize the fold; and (**C**) The mainly chain have four domain which B-domain (yellow), C-domain (green), A-domain (cyan) and D-domain (blue).

The 3D structure of yak IGF2 was predicted using homology structure modeling on a Swiss-model server [20]. The 3D structure of human IGF2 (PDB ID 1iglA) with 95.38% sequence identity was used as a template to predict the 3D structure of yak IGF2. The result revealed overall folding and secondary structures highly similar to those of *Homo sapiens* (Figure 6A). The 3D structure of yak IGF2 was a single chain that includes three α-helixes which contains small disulfide-rich folds (Figure 6B). Yak IGFs consisted of so-called B, C, A and D “domains” (Figure 6C) (regions of sequence in order from the *N* to the *C* terminus) [21]. The B and A domains were similar in structure to the equivalent domains in insulin. These domains contained three α-helices: Helix 1 (Gly35-Val44 of IGF2) was in the B domain, whereas helix 2 (Glu68-Phe72) and helix 3 (Ala78-Tyr83) were both located in the A domain in which two smaller parallel α-helices were the key structure features of these proteins. The C and D domains were highly flexible. The structure was held together by three disulfide bonds (Cys33-Cys71, Cys45-Cys84 and Cys70-Cys75). Major determinants for IGF-1R and IR-A binding were located at the end of the B domain (including Arg48, Phe50 and Tyr51 residues).

#### 2.1.5. Similarities between Structure of Yak IGF2 and Other Mammalian IGF2

**Figure 7.**
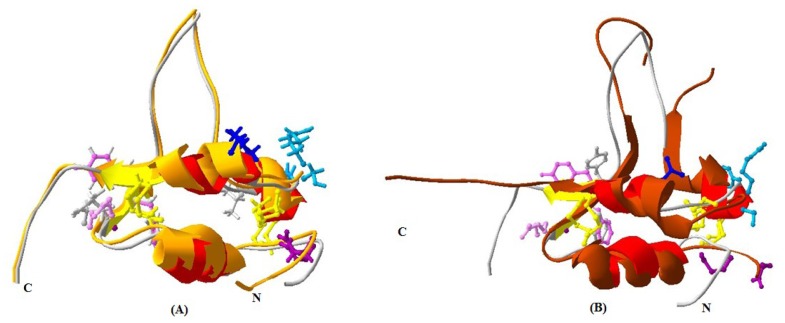
Superimposed 3D structure: Tianzhu white yak IGF2 (red) with human IGF2 (Ginger) (**A**) and human IGF1 (brown) (**B**). Binding sites were shown as rodlike molecules. The superimposition indicated very high similarity between the structures of yak IGF2 and the 3D structure of human IGF2. Binding sites were shown as rodlike molecules for yak IGFBP (modena), yak IGF-receptor and insulin-receptor and IGFBP (mauve) and yak IGF-receptor (blue); human IGFBP (modena), type 1 human IGF-receptor and insulin-receptor (white), type 2 human IGF receptor and IGFBP (grey), human IGF-receptor (blue). The view of IGF2 revealed three disulfide bonds (yellow, rod-like) which stabilize the fold.

**Figure 8.**
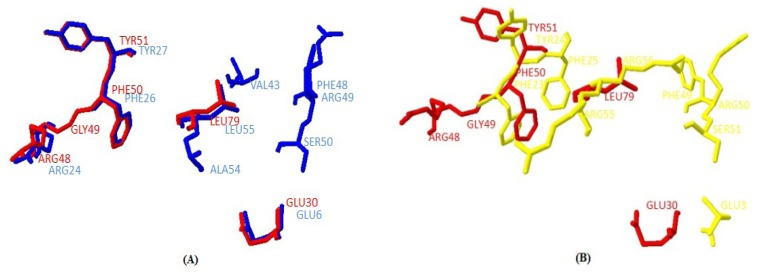
Comparison of the binding site residues of Tianzhu white yak IGF2 (red) with *Homo sapiens* IGF2 (blue) (**A**) and *Homo sapiens* IGF1 (yellow) (**B**). The binding site of the IGF from the two species and two amino acid sequence contains the highly conserved Phe and Tyr residues. This comparison indicated very high similarity between the predicted binding sites of yak IGF2 and human IGF2.

The main secondary structure elements in yak IGF2 were α helices encompassing residues 35 to 44, 55 to 57, 61 to 72 and 77 to 82. A small anti-parallel β-sheet was formed by residues 49 to 51, and 83 to 85 (Figure 6A). The similarities between yak IGF2 and human IGF2 were studied by superimposing their structure in the PyMOL program (http://pymol.org) [22]. The overall folds of predicted yak IGF2 structure were highly similar to human IGF2 and IGF1 (Figure 7A,B). The quality of the structural homology was calculated using PDBe Fold on the EMBL-EBI server [23] (Table 3). When the 3D structure of yak IGF2 was aligned with that of human, 65 residues were aligned out of 67 input residues. Sequence identities between yak IGF2 *vs.* human IGF2 and human IGF1 were 95% and 80%, respectively. The overall RMSD (root mean square deviation) between yak IGF2 and human IGF2 structure was 0.09 (Table 3). The major structural difference was found in the binding site (Figure 7A,B and Figure 8A,B). The flexible loop in yak and human IGF2 were superimposed fairly well (Figure 7A). The *Q*-score is 0.97 (closed to 1.0 means identical structure) when compared with human IGF2. Similarly, the superimposed yak IGF2 structure with human IGF1 had a *Q-score* of 0.45. The *p*-score was used to evaluate the significance of structure similarity. Superimposition of yak IGF2 with human IGF2 and IGF1 had *p*-scores of 10.8 and 1.8, respectively (Table 3). The *Z*-scores of yak IGF2 structure superimposition with human IGF2 and IGF1 were 9.7 and 3.8, respectively (Table 3). Therefore, the values of *Q*-scores, *p*-scores and *Z*-scores indicated that the structure of yak IGF2 was highly similar to the structure of human IGF2.

**Figure 9.**
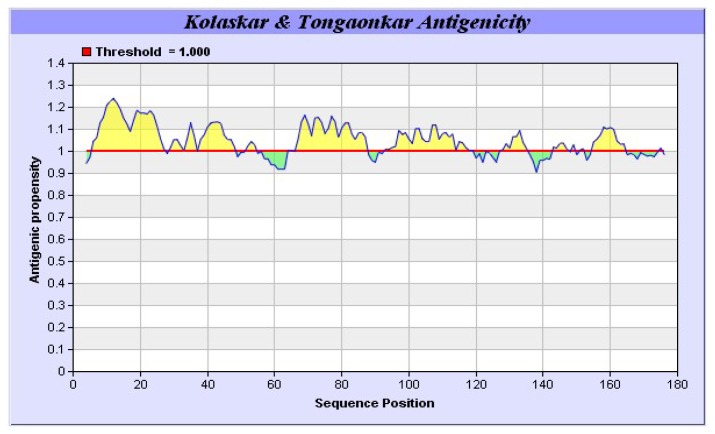
Antigenic properties of yak IGF2 predicted by using Kolaskar and Tongaonkar method. Six potential antigenic peptides having more than 1.0 antigenic propensity and nine or more amino acids in lengths were predicted.

**Figure 10.**
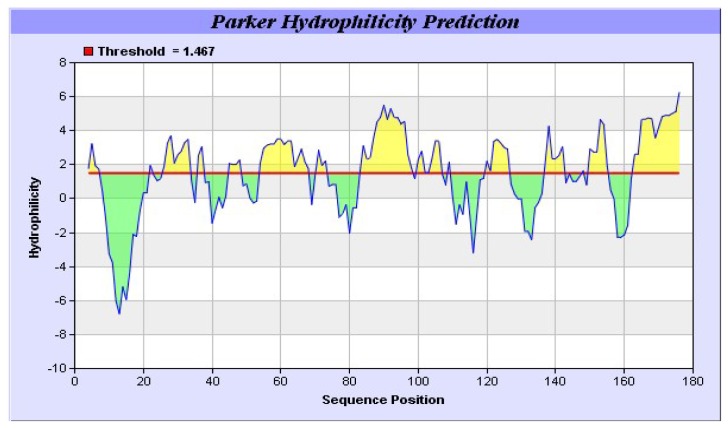
Predicted hydrophobic regions of yak IGF2. The hydrophobic residues forming the binding interface were directed under the threshold value and represented by the residues L32, L37, V38, V44, I59, I67 and LALL (77–80). Transmembrane residues were directed under the threshold line and represented as SVLVLLAFLAFASCCYAA (8–25).

**Figure 11.**
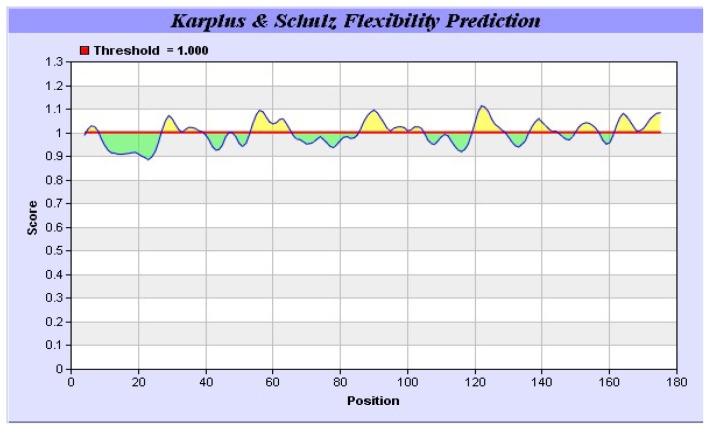
Predicted flexibility regions of yak IGF2. The flexibility residues forming a flexibility loop were directed above the threshold value and represented by the residues RPSSRINRRSRG (54–65).

It was also predicted that yak IGF2 was highly antigenic since the majority of the protein surface was exposed to the aqueous medium. At least six potential antigenic peptides with more than 1.0 antigenic propensity (Figure 9) and nine or more amino acids in lengths were predicted. The antigenic amino acid sequences and its position were listed in Table 4. The hydrophobic regions of yak IGF2 were also predicted (Figure 10). One binding interface could be formed by the hydrophobic residues based on the threshold value and represented by the residues L32, L37, V38, V44, I59, I67 and LALL (77–80). Transmembrane residues based on the threshold line were represented as SVLVLLAFLAFASCCYAA (8–25). Flexibility residues forming A flexibility loop was formed by the residues RPSSRINRRSRG (54–65) based on the threshold value (Figure 11).

#### 2.1.6. Expression of *IGF2* Gene by Real Time PCR in Tianzhu White Yak

**Figure 12.**
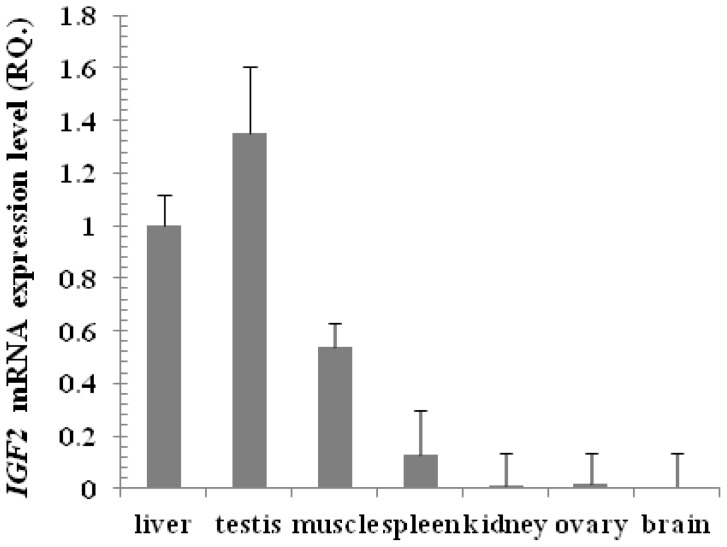
Expression of yak IGF2 was detected by Real time PCR from different tissues. The results were expressed relative to liver as calibrator and using the *GAPDH* as housekeeping gene.

The mRNA expression level of *IGF2* was examined using Real time PCR in nine yak tissues. The primers were designed to amplify 194 base pairs and the experimental conditions were adjusted for the best annealing between primers and cDNA, to eliminate primer dimer, self-dimer or hairpin forms and to amplify only one band representing part of the yak *IGF2*. The expression of *IGF2* in the liver was taken as a reference sample (calibrator) and the expression of *GAPDH* as a housekeeping gene (endogenous control). The relative expression of yak *IGF2* in the testis, muscle, spleen, kidney, ovary and brain were compared with that of liver. The expression level in testis was 1.35 fold (135%) higher than in liver, followed by muscle (53%), spleen (12%), kidney (1%), ovary (1%) and brain (0.6%) relative to liver (100%), respectively (Figure 12). However, the expression of *IGF2* was not detected in heart and lung.

### 2.2. Discussion

The yak lives in a tough but important environment for the local residents and research. Study of its growth mechanism is of considerable significance in yak breeding, cold and plateau resistance, and also in mechanisms of material absorption and deposition.

Full sequence of yak IGF2 was amplified in the present study, yielding a cDNA fragment of 1060 bp covering the coding region of yak *IGF2* using a primer set spanning the gene (Figure 1). This sequence covered promoter, and terminator parts of the 5′ and 3′ untranslated region and a 540 bp open reading frame, comparable with most mammalian species (Figure 2). The predicted product was a protein of 179 amino acid residues of 19.68 kDa, which matched with IGF2 sequences of mammalian species in GenBank. This sequence was submitted to the NCBI GenBank database and received accession number KF682139.

The primary sequence homology of IGF2 between yak and other mammalian species was greater than 95% (Table 2). This confirms the specificity of the primer set and the position of yak IGF2 in the IGF family. The secondary structure (Figure 5) analysis showed that yak IGF2 was mainly a single amino acid sequence, which included a signal peptide, 3 α-helixes and 2 anti-parallel sheets in the 27–91 position. A central α-helix of the B domain and two smaller and parallel α-helices in the A domain composed the key structural features of IGF2; the α-helical segments were stabilized by three strictly conserved disulfide bonds (Figure 6) of the known IGF2 class. These were the typical characteristic of the IGF family [21]. The conserved flexibility residues’ sequence formed the “flexibility loop”, where the active site was located from the residue 54 to 65, characterized with flexible amino acids 54-RPS(A)SR(L)IN(S)RRSRG-65. The 56, 58 and 60(S, R, N) residues have been replaced by A, L and S in other mammalians, respectively. Based on the reported characteristics of the structure of IGFBP, IGF-receptor and insulin-receptor [24], we predicted that yak IGFBP was binding to Glu30, Arg48, Gly49, Phe50 and Try51 residues, yak insulin-receptor binding to Arg48, Phe50 and Try51 residues, yak IGF receptor binding to Arg48, Phe50, Try51 and Leu79 residues. There were three shared residues (at Arg48 Phe50 and Try51) binding to yak IGFBP, yak IGF-receptor and yak insulin-receptor. Proteins with similar amino acids sequences have a tendency to adopt similar 3D structures. Therefore, the 3D structure of the putative yak IGF2 was predicted by homology structure modeling in Swiss-model server according to the recently published *Homo sapiens* IGR2 NMR-derived structure [25]. It was reported that the mature IGF was a single chain polypeptide composed of four domains convolved together by hydrophobic and some electrostatic interactions [26]. Three disulfide bonds formed small disulfide-rich folds responsible for the stability of yak IGF2; the disulfide bond formation reduced the conformational entropy. The longer the length between disulfide bonded cysteine, the larger the entropic contribution to the stabilization of the folded protein structures [27].

In mammalians, IGF2 can combine with three kinds of conservative receptor proteins (Figure 7A,B). The overall quaternary structure, folding, and topology were quite similar to *Homo sapiens* IGF2 (Figure 7). The hydrophobic residues forming the yak IGF2 binding interface were directly under the threshold value and were represented by L32, L37, V38, V44, I59, I67, LALL (77–80), A85 and A78. The transmembrane residues were exactly under the threshold line and represented as SVLVLLAFLAFASCCYAA (8–25).

The result from the superimposition of yak IGF2 compared with human IGF1 and IGF2 indicated that yak IGF2 was well superimposed with human IGF2 in 3D structure. A *Q*-score of 0.45 and 0.97 was indicated when compared to human IGF1 and IGF2 (The *Q*-score indicated the level of similarity in 3D structure, from 0 to 1, and 0 indicates no similarity while 1 refers to identitical [28]). The *p*-scores were 1.8 and 10.8, and the *Z*-scores were 3.8 and 9.7 (*Z*-score exceeds 3.5 means the structures are similar to each other), which also indicate a higher similarity of yak to human (Table 3).

Real time PCR data suggested that yak testis has the highest expression of *IGF2*, followed by liver, muscle, spleen, kidney, ovary and brain. This result showed that the expression of yak *IGF2* may be related to the reproductive axis and regulate the signaling pathway in reproduction to control *IGF2* gene expression and to promote bone and muscle growth, and then increase the meat production. Although this paper provides detailed information about yak *IGF2* sequences and its structure, more detailed studies are required to explore the role of yak *IGF2* in gene regulation, function, response to muscle growth, and action at the molecular level. The yeast two-hybrid system will be used for further studies of yak *IGF2* function. RNAi and conditional gene knockout technology will also be applied to study the muscle growth mechanism using yak as an animal model.

## 3. Experimental Section

### 3.1. Brief Introduction of Methods

The PCR primers and RACE primers were designed based on the conserved sequence homology from *Bos Taurus, Sus scrofa* and *Ovis aries* available from National Center for Biotechnology Information (NCBI) for initial screening. Bioinformatics software and website were used for the analysis of the biological sequence information. The related software and website were illuminated in Table 5. Real time PCR was used for detection of the *IGF2* gene expression.

### 3.2. Samples and Materials

Fresh liver, kidney, spleen, lung and testis tissues from adult male yaks were obtained immediately after slaughtering from the Slaughterhouse of Tianzhu County (Wuwei City, China). Yak tissues were immediately stored at −80 °C.

### 3.3. RNA Isolation and cDNA Synthesis

Total RNAs were extracted from yak liver tissues by using RNAiso Plus (Takara Code No. 9109, Dalian, China) following manufacturer’s instructions and was further used for cDNA synthesis. RNA was quantified by NanoDrop-8000 (Thermo, Waltham, MA, USA) and RNA integrity was assessed on denaturing formaldehyde agarose gel (1%) electrophoresis (Biowest Regular Agarose, No. 111860, Castropol, Spain). Two micrograms of total RNAs were subjected to reverse transcription to single-stranded cDNA using BioTeke Thermo RT Kit (Bioteke, Cat No. PR6601, Beijing, China), the reverse transcription PCR reaction was 48 °C for 50 min, followed by 70 °C for 10 min. The total 20 μL cDNA synthesis reaction contained 2 μL total RNA, 1 μL Oligo dT or Random Primer (50 μM), dNTP Mixture (10 μM), Thermo M-MLV (200 U/μL), RNase Inhibitor (40 U/μL), 4 μL 5× first-strand buffer and 10 μL of ultrapure millipore water (Invitrogen, Carlsbad, CA, USA).

### 3.4. Synthesis and Confirmation of Partial cDNA of *IGF2* Gene

The *IGF2* primers were designed based on conserved sequence homology from *Bos taurus and Sus scrofa* available from NCBI for initial screening. The special primers used for initial screening and obtaining partial *IGF2* cDNA of Yak were Forward 5′-CTGGAAGCAGTCCACCCAG-3′ and Reverse 5′-TTCTGGTGTTGCTAAAAAGCC-3′. The PCR reaction mixture (30 μL volume) contained 2 μL of template cDNA, 1.5 μL (10 μM) of each primers set, 15 μL 2× Power Taq PCR MasterMix, (BioTeke Cat No. 71182, Dalian, China) and 10 μL of ultrapure millipore water and was carried out by Bio-Rad PCR (BIO-RAD, Hercules, CA, USA). The PCR amplification conditions were as follows; initial denaturation at 95 °C for 5 min followed by 35 cycles at 95 °C for 1 min, 52 °C for 1 min and 72 °C for 1 min. Final extension step was carriedout at 72 °C for 10 min. The products of PCR reactions were electrophoresed on an agarose gel (1.5%) using DNA Ladder DL2000 (Gstar, Cat No. TZM 122-01, Chendu, China). The 544 bp fragment from the PCR reaction was eluted and purified using BioTeke Gel Extraction kit (BioTeke Lot No. G51101, Beijing, China). Purified fragments were quantified and validated on agarose electrophoresis before sequencing. PCR fragments were sequenced by BGitech Company, Beijing, China.

### 3.5. Rapid Amplification of cDNA Ends (RACE), Cloning and Sequencing

Rapid-amplification of cDNA ends (RACE-PCR) was used to identify and isolate the 5′- and 3′-end of *IGF2* using RACE kits (Invitrogen No.18373019/18374058, Carlsbad, CA, USA). Yak total RNA was annealed with 5′- and 3′-end adaptor primers, and reversely transcribed respectively to respective 5′- and 3′-cDNA. The resulting single stranded 5′- and 3′-cDNA were then used as templates in PCR. For 5′ RACE, the 5′-end adaptor primer (forward primer 5′-CGCAGACAGGACGGTACAGGGATTTCA-3′) and *IGF2* 5R (reverse primer 5′-CTCACTTCTAATCGCTGGATGCCTTGGA-3′) were used. For 3′ RACE, *IGF2* 3F primer (forward primer 5′-TCCAAGGCATCCAGCGATTAGAAGTGAG-3′) and the 3′-end adaptor primer (reverse primer 5′-TCTGAAATCCCTGTACCGTCCTGTCTGC-3′) were used. The cycling program was set for five cycles of 94 °C, 5 min; 5 cycles of 94 °C 15 s, 70 °C 15 s, 72 °C 3 min; 5 cycles of 94 °C 15 s, 68 °C 15 s, 72 °C 3 min; 5 cycles of 94 °C 15 s, 65 °C 15 s, 72 °C 3 min; 25 cycles of 94 °C 15 s, 60 °C 15 s, 72 °C 3 min; 1 cycle of 72 °C, 5 min. The RACE PCR products were purified and cloned into pMD-18 vector (Takara Code No. 6011, Dalian, China) to confirm the sequences of the 5′- and 3′-end, respectively. The full-length cDNA PCR product was cut with the restriction enzymes, purified, and ligated with T4 DNA ligase into a pMD-18 vector. Competent *E. coli* DH5α cells were transformed with the plasmids and selected by means of antibiotic resistance. The sequence of the inserted full-length cDNA was confirmed with DNA sequencing.

### 3.6. Analysis and Alignment of cDNA Sequence

The sequence of yak *IGF2* gene was analyzed for nucleotide, protein translation, sequence alignment and comparisons with other mammalian species [37]. The sequenced DNA fragment was translated using EditSeq of DNASTAR program [38] and the deduced yak IGF2 amino acid sequence was compared in the NCBI Protein Database using the BLASTP algorithm [39]. DNA and protein homologies were analyzed by using NCBI BLAST program via NCBI web-servers [40]. Signal P 4.1 server was used to predict the presence and location of signal peptide cleavage sites in amino acid sequences from yak IGF2 (http://www.cbs.dtu.dk/services/SignalP/) [41]. Bioinformatics Toolkit Quick2D program was used to predict the transmembrane helices in proteins [42]. The method reported by Parker *et al.* [43] was used to analysis the hydrophilic/hydrophobic of IGF2 in Tianzhu white yak. An amino acid scale was defined by a numerical value assigned to each type of amino acid. The most frequent scales were hydrophobicity or hydrophilicity scales and the secondary structure conformational parameters scales, but many other scales based on different chemical and physical properties of the amino acids also exist [44].

### 3.7. Protein and mRNA Secondary Structure Prediction

Bioinformatics Toolkit Quick 2D program [35] was used to construct the secondary structure of yak *IGF2* mRNA sequence with the free energy minimization algorithm. RNA tertiary structures was characterized by secondary structural elements based on hydrogen bonds within the molecule that form several recognizable “domains” of secondary structure like stems, hairpin loops, bulges and internal loops. A poly(A) tail and poly(A) signal for the yak *IGF2* mRNA was predicted based on the free minimization algorithm of CLCbio (Dusseldorf, Germany) [45]. The predicted molecular weight, isoelectric point (pI) and charges of the protein IGF2 were estimated.

### 3.8. Multiple Sequence Alignment and Phylogenetic Analysis

The amino acid sequence of yak cIGF2 was used as template to identify homologous mammalian sequences in PSI-BLAST. Nine homologous sequences from different mammals were used for multiple sequence alignment by ClustalW on MEGA5.1 software [46]. The output of Multiple Sequence Alignment was color-coded according to their identity. The phylogenetic tree was constructed using amino acid sequences of yak IGF2 from all nine mammalian species by the BLOSUM62 from MAFFT Multiple Sequence Alignment [19].

### 3.9. Yak *IGF2* Protein 3D Structure Prediction

The 3D structure of yak IGF2 was predicted using a Swiss-model server base on homology structure model [47]. The similarities between modeled human IGF2 and IGF1 structure, the catalytic and enzymatically important residues and DNA backbone interaction residues in IGF2 were superimposed on IGF2 using Pymol software (delino Scientific, New York, NY, USA) [48]. The quality of the superimposed 3D structures was assessed using PDBe on the EMBL-EBI server [23].

The antigenic properties of yak IGF2 were predicted according to the methods of Kolaskar and Tongaonkar [49]. The antigenicity of peptides was predicted with the more than 1.0 antigenic propensity threshold and more than six amino acid residues. The hydrophobicity of yak IGF2 was calculated according to the method of Parker [34]. The flexibility of yak IGF2 was predicted with the method of Karplus and Schulz [50].

### 3.10. Real Time PCR Assays for *IGF2* Gene Expression in Yak

The gene expressions level of *cIGF2* was detected by quantitative Real time PCR. 200 ng total RNA was used for the synthesis of single-stranded DNA (cDNA) with BioTeke Thermo RT Kit (Bioteke, Cat No. PR6601, Beijing, China). PCR was performed using 2 μL of cDNA in a 25 μL reaction volume with ABI7300 (ABI system, Foster, CA, USA) Real time System. SYBR Premix Ex Taq™ II (Takara, Dalian, China) and specific primers were used in each reaction. The sense and antisense primers were designed with the Primer 5.0 based on the *IGF2* cDNA sequences. Primer sequences used were as follows: sense: 5′-ATACCCCGTGGGCAAGTTC-3′, antisense: 5′-ACGACACTTTGGCTTTGGCTCACTTCTAATC-3′, product size 138 bp), *GAPDH* (sense: 5′-AAGTGGGGTGATGCTGGTG-3′, antisense: 5′-GCTGACAATCTTGAGGGTGTTG-3′, product size 189 bp, GenBank No. NM_008084). The expression of *IGF2* in the liver was taken as a reference sample (calibrator) and the expression of *GAPDH* as a housekeeping gene (endogenous control). ABI7300 PCR system was used, and the 2-step standard procedure for amplifying the samples was applied. Stage 1: for denaturation, Reps 1, 95 °C for 30 s. Stage 2: PCR reaction, Reps 40, 95 °C for 5 s and 60 °C for 31 s. The final step is the dissociation stage. All PCR reactions were performed in triplicate. The results were calculated applying the 2^−ΔΔ^*^Ct^* method [51].

## 4. Conclusions

The putative full sequence of yak IGF2 was successfully cloned for the first time, and its sequence and predicted product structure was found to be very similar to human IGF2. As a specific species and a model animal living in the highland with low oxygen content, research of IGF will greatly facilitate resistance studies, and benefit yak breeding in similar areas. There were several binding sites in yak IGF2 that can accept different receptors, which play crucial roles in regulating skeletal muscle growth and differentiation, as well as maintaining homeostasis. The present work represents initial research into yak IGF2; future studies will be aimed at the clarifation of the functions of IGF2 and the mechanisms of bone and muscle growth.

## Figures and Tables

**Table 1.** Predicted chemical composition of the cloned fragment of IGF2.

Amimo Acid	Number Count	% By Weight	% By Frequency
Ala (A)	20	7.22	11.17
Arg (R)	18	14.28	10.06
Asn (N)	1	0.58	0.56
Asp (D)	9	5.26	5.03
Cys (C)	8	4.19	4.47
Gln (Q)	5	3.26	2.79
Glu (E)	9	5.90	5.03
Gly (G)	12	3.48	6.70
His (H)	2	1.39	1.12
Ile (I)	5	2.87	2.79
Pyl (O)	0	0	0
Leu (L)	19	10.92	10.61
Lys (K)	7	4.56	3.91
Met(M)	1	0.67	0.56
Phe (F)	9	6.73	5.03
Pro (P)	9	4.44	5.03
Ser (S)	17	7.52	9.50
Thr (T)	12	6.16	6.70
Trp (W)	1	0.95	0.56
Tyr (Y)	6	4.97	3.35
Val (V)	9	4.53	5.03
Sec (U)	0	0	0

**Table 2.** Comparison of Tianzhu white yak (*Bos grunniens*) IGF2 and other IGF2 proteins from various, mostly similar, mammals. The comparison included number of amino acid sequence, percent identity, *E*-value, isoelectric point (pI) and subunit molecular weight.

IGF-2	(NCBI Reference Sequence)	No. of Residues	Total Score	Coverage (%)	Identity (%)	Positive (%)	*E*-Value	pI	Mean Value	Gaps (%)
*Bos grunniens*	KF682139	179	364	100	100	100	2e- 134	8.82	19.68	0
*Bos taurus*	AY957981.1	179	362	100	99	99	4e- 133	8.65	19.64	0
*Bubalus bubalis*	KC107769.1	135	278	75	100	100	3e- 101	9.23	13.77	0
*Bos primigenius*	AF283002.1	149	305	83	100	100	2e- 111	10.50	15.11	0
*Ovis aries*	M89788.1	179	331	100	96	97	5e -121	8.49	19.62	0
*Capra hircus*	DQ645739.1	179	350	100	96	97	9e -129	8.49	19.65	0
*Cervus elaphus*	EF177491.1	179	335	100	92	94	1e -122	8.67	19.72	0
*Sus scrofa*	HQ450757.1	181	289	98	84	88	2e -104	9.99	20.31	0
*Homo sapiens*	XM005252900.1	180	309	100	84	88	1e -112	9.34	20.14	0

**Table 3.** Pairwise alignment between predicted structure of Tianzhu white yak IGF2 and *Homo sapiens* IGF2 and IGF1. RMSD (root mean square deviation), PDB (protein data bank).

Structure	(PDB)	No. of Residues	Aligned Residues	RMSD	*Q*-Score	*p*-Score	*Z*-Score
**Predicted yak IGF2**	human IGF1 (1H02)	64	49	1.61	0.45	1.8	3.8
**Predicted yak IGF2**	human IGF2 (1IGL)	67	65	0.09	0.97	10.8	9.7

**Table 4.** The antigenic amino acid sequences and its position.

NO.	Start Position	End Position	Peptide	Peptide Length
**1**	6	27	GKSVLVLLAFLAFSCCYAAYR	22
**2**	29	48	SETLCGGELVDTLQFVCGDR	20
**3**	67	87	VEECCFRSCDLALLETYCATP	21
**4**	93	119	DVSASTTVLPDDVTAYPVGKFFQYDIW	27
**5**	127	135	RRGLPAFLR	9
**6**	155	164	HRPLIALPTQ	10

**Table 5.** Brief introduction of the softwares and websites.

Function	Software and website
To search nucleotide sequence	http://www.ncbi.nlm.nih.gov/nuccore/ [29]
To find conserved sequence	MEGA 5.1/DNAstar/DNAMAN [30]
To design primers in the conserved sequence	Oligo 7.0/Primer Premier 5 [31,32]
To find open reading frame	http://www.ncbi.nlm.nih.gov/gorf/gorf.html [33]
To analysis amino sequence	PROTEAN program/DNAMAN [17,30]
To analysis identity of amino acid	http://blast.ncbi.nlm.nih.gov/Blast.cgi [34]
To alignmultiple sequence	MAFFT Multiple program/Sequence Alignment [18]
To analysis phylogenetic	MAFFT Multiple program/Sequence Alignment/MEGA 5.1 [18]
To predict secondary structure	Bioinformatics Toolkit Quick2D program [35]
To predict 3D structure	http://swissmodel.expasy.org/ [20]
To analysis 3D structure	Swiss-PDB Viewer program [20]
To predict antigenic properties	http://imed.med.ucm.es/Tools/antigenic.pl [36]
